# Development of a TaqMan Array card to target 21 purulent meningitis-related pathogens

**DOI:** 10.1186/s12879-019-3856-z

**Published:** 2019-03-28

**Authors:** Chengna Zhao, Xi Wang, Chao Zhang, Bing Liu, Hongbo Jing, Lihua Ming, Hua Jiang, Yuling Zheng, Peng Liu, Gang Liu, Yongqiang Jiang

**Affiliations:** 1 Anhui Medical University, Hefei, China; 2 State Key Laboratory of Pathogen and Biosecurity, Beijing Institute of Microbiology and Epidemiology, Beijing, China; 3 Department of Infectious Medicine, Beijing Children’s Hospital, Capital Medical University, National Center for Chidren’s Health, Beijing, China; 4 Shunyi District Center for Disease Control and Prevention, Beijing, China; 5 Chest Hospital of Xinjiang, Urumqi, China

**Keywords:** Purulent meningitis, TaqMan Array card, Real-time PCR, Cerebrospinal fluid culture, Detection method

## Abstract

**Background:**

Purulent meningitis (PM) is a serious life-threatening infection of the central nervous system (CNS) by bacteria or fungi and associated with high mortality and high incidence of CNS sequelae in children. However, the conventional cerebrospinal fluid (CSF) culture method is time-consuming and has a low sensitivity.

**Methods:**

Our study developed a real-time PCR-based purulent meningitis-TaqMan array card (PM-TAC) that targeted 21 PM-related pathogens and could produce results within 3 h. Primers and probes were adapted from published sources possibly. The performance of them were evaluated and optimized and then they were spotted on TAC.

**Results:**

The PM-TAC showed a sensitivity and specificity of 95 and 96%, respectively. For all of the 21 targeted pathogens, the PM-TAC assay had a LOD ranging from 5 copies/reaction to 100 copies/reaction, an intra-assay variation of 0.07–4.45%, and an inter-assay variation of 0.11–6.81%. Of the 15 CSF samples collected from patients with PM after empiric antibiotic therapies, the positive rate was 53.3% (8/15) for our PM-TAC assay but was only 13.3% (2/15) for the CSF culture method. Of the 17 CSF samples showing negative CSF culture, the PM-TAC assay identified a case of *Neisseria meningitidis* infection. Furthermore, all of the 10 CSF samples from patients without CNS infection showed negative for the PM-TAC assay.

**Conclusions:**

Our PM-TAC assay also demonstrated that the pathogen loads in the CSF samples correlated with the severity of PM. Thus, the PM-TAC may be helpful to improve the prognosis of PM and clinical outcomes from antibiotic therapies.

**Electronic supplementary material:**

The online version of this article (10.1186/s12879-019-3856-z) contains supplementary material, which is available to authorized users.

## Background

Purulent meningitis (PM), which is often caused by bacterial or fungal infection, is a serious disease of the central nervous system and particularly life-threatening for children and newborns. The disease is characterized by acute onset, high fever, severe headache, vomiting, stiff neck, and high disability and mortality rates. But in infants and young children, these “classical “signs are often absent, they may be poor feeding, lethargy, disorientation or reduced conscious level. Approximately 10 to 20% of PM survivors develop post-PM neurological sequelae [1]. The common sequelae of PM include hydrocephalus, cerebral hemorrhage, epilepsy, paralysis, blindness, and mental retardation [2, 3]. Thus, timely and accurate treatments are critical to improve PM prognosis, and effective treatments for PM depend on an accurate and rapid identification of the causative bacteria or fungus [4, 5].

The commonly recognized gold standard to identify the causative bacteria for PM is the cerebrospinal fluid (CSF) culture method [6, 7]. However, the CSF culture method has many limitations. For instance, the procedure is too complex; the time for an affirmative result is too long (usually longer than 48 h); the method often fails if patients receive empiric antibiotic therapies [8, 9].

The direct Gram staining method can provide results rapidly and confirm a diagnosis of bacterial infection in 60–90% of patients, and a confirmed diagnosis from Gram staining depends on the bacterial concentration in CSF samples [7]. The Gram staining method may fail when the bacterial concentration is too low in CSF samples, and the sensitivity of the Gram staining method also varies widely for different microorganisms [10]. Although CT may be useful to diagnose PM, a low accessibility to CT scanners and the requirement of further laboratory tests limit its application [11]. The results of the method of MALDI-TOF MS were usually affected by culture media, cultivation conditions, or incubation times [12, 13]. A regular PCR combined with gel electrophoresis has a low sensitivity. The sequencing method is usually time-consuming, has a complex protocol, and is expensive [14–16]. Luminex Verigene, a high-throughput platform, can detect multiple pathogens from one sample simultaneously but requires a relatively long time to collect results [17]. In 2015, the United States Food and Drug Administration approved the first multiplex PCR kit (FilmArray® system) as an auxiliary diagnostic method to identify causative pathogens of meningitis and encephalitis. The FilmArray® system [18, 19] can simultaneously detect 14 pathogens (6 bacteria, 7 viruses, and *C. neoformans/C. gattii*) from CSF samples in approximately 1 h. However, the system is very expensive ($305–$453/sample and $22–$32/pathogen) [20, 21].

A TaqMan array card (TAC), which combines the real-time PCR technology and microfluidics high-throughput technology, appears superior to those methods. A TAC has 384 wells on a microfluidic chip and thus can detect up to 48 targeted pathogens simultaneously using a small amount of a sample. The TAC technology has been used to identify respiratory pathogens [22], enteropathogens [23], bioterrorism pathogens [24, 25], neonatal infection [26], and drug resistance mutations [27]. Liu and colleagues compared the performance of a TAC, multiplex real-time quantitative PCR, and the PCR-Luminex platform in the identification of enteric pathogens [28], and they found that the TAC showed the best sensitivity and specificity. TAC assays were used to identify the causative microorganisms of respiratory disease outbreaks in the United States [29]. Although TAC systems have been used successfully to identify pathogens, a comparison of the sensitivity of a TAC versus the CSF culture method to identify pathogens in CSF samples from patients receiving empiric antibiotic treatments is still lacking. Moreover, whether TAC results could indicate PM severity remains unclear. Here, we aim to fill these knowledge gaps. In the current study, we developed a high-throughput PM-TAC, compared the sensitivity of the PM-TAC versus the CSF culture method to detect pathogens from CSF samples of children with PM after using antibiotics, and explored whether PM-TAC results could indicate PM severity.

## Methods

### The design of the PM-TAC

**Fig. 1:**
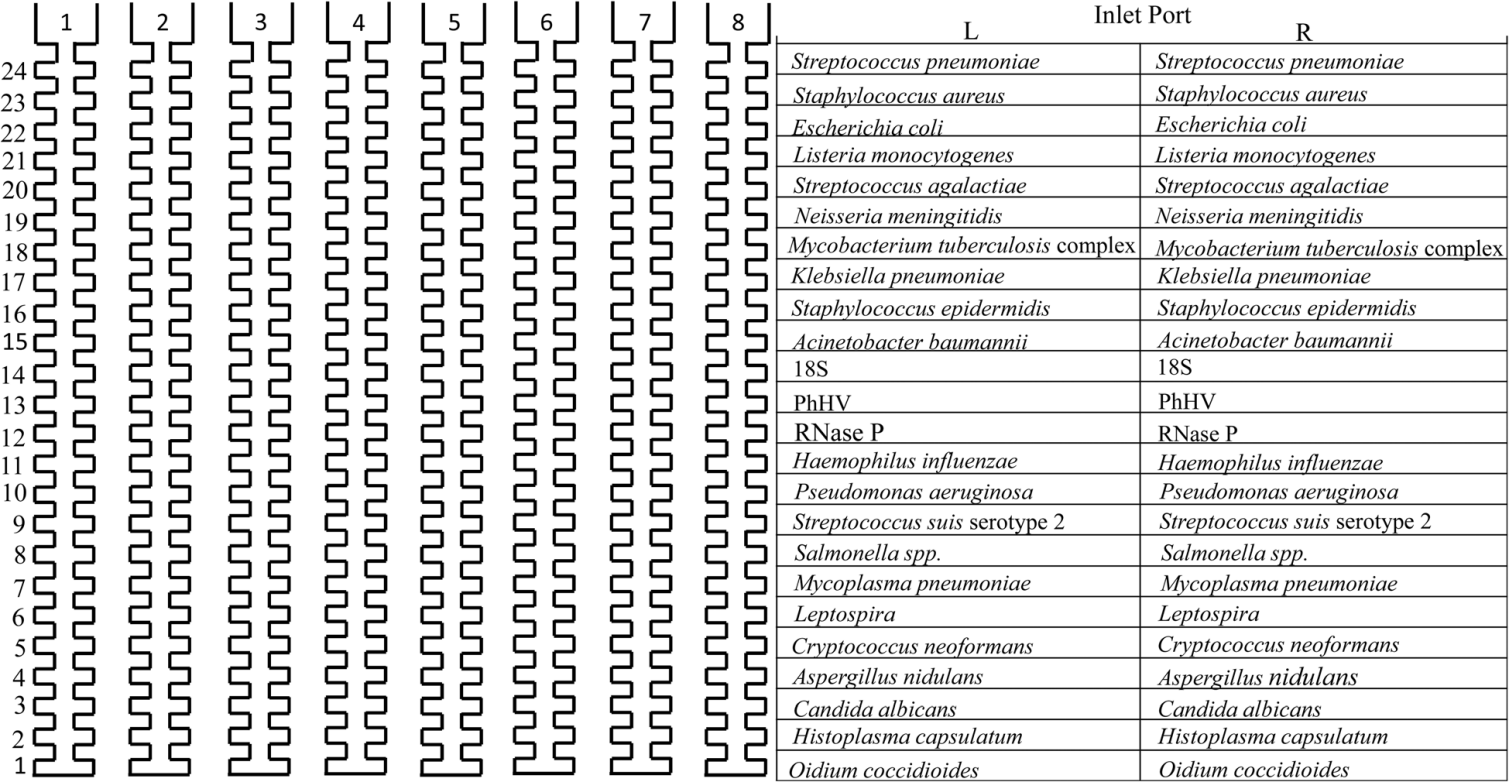
Configuration of the Purulent Meningitis-TaqMan Array Card

The PM-TAC targets 21 pathogens (Fig. 1), including 16 bacteria and 5 fungi (Additional file 1: Table S1). The primer and probe sequences for the majority of the targeted pathogens were originally from previous publications except that the sequences for *Histoplasma capsulatum* and *Oidium coccidioides* were designed in the current study. A single gene was targeted for each individual pathogen and every target was duplicated. The BLAST from the NCBI database, Clustal, and Primer Express (Life Technologies, Carlsbad, CA) were used to design and evaluate the primer and probe sequences. The sequences were optimized for the universal cycling conditions. All of the primers and probes were validated by real-time PCR assays using positive-control plasmids or pathogen genomic DNA. An off-target DAN template contains DNA template sequences of all the target pathogens on the TAC except the DNA template sequences of one pathogen that is targeted by its specific primer/probe pair. An on-target DNA template contains the DNA template sequences of one pathogen that is targeted by its specific primer/probe pairs. The DNA sequences of primers and probes are described in Additional file 1: Table S1. The universal formula for TAC assay was used. The final concentrations of each primer and probe were 900 nM and 250 nM, respectively.

### Control plasmid construction

**Fig. 2:**
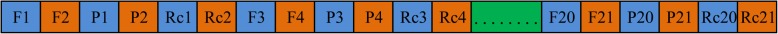
The design the oligonucleotide inserted in the control plasmid. The oligonucleotide includes the sequences of primers and probes of the 21 targeted pathogens. F: forward primer; P: probe; Rc: the reverse complement of reverse primer. F1 is referring to the forward primer sequence of pathogen 1; F2 is referring to the forward primer sequence of pathogen 2; P1 is referring to the probe sequence of pathogen 1

A long oligonucleotide containing the sequences of primer and probe sequences of all of the targeted pathogens was first synthetized by Sangon Biotech. The oligonucleotide included the forward primer sequences, probe sequences, and the reverse complement of the reverse primer sequences of the 21 targeted pathogens. The detailed design of the oligonucleotide is illustrated in Fig. 2. The oligonucleotide was used as an artificial template [30]. The oligonucleotide was inserted into the pMDTM19-T vector using the pMDTM19-T Vector Cloning Kit (TAKARA). The recombinant pMDTM19-T construct was used as a control plasmid.

### Evaluation of the PM-TAC

A dose effect of the control plasmid on the PM-TAC assay was analyzed. We also prepared an artificial cerebrospinal fluid (ACSF) according to a previous description (43). To estimate the accuracy and LOD of the PM-TAC, the control plasmid was added to the ACSF. To determine the accuracy, two doses of the control plasmid were used for each targeted pathogen: a low dose, which was the LOD estimated from the real-time PCR assay, and a high dose, which was a dose of 100 times of the LOD. To estimate the intra-assay variations, 8 replicates were used for each dose. To estimate inter-assay variations, 4 replicates were used for each dose and 5 batches of PM-TAC were used, and the assay for each batch was conducted on different days. To determine the LOD of the PM-TAC, several doses of the control plasmid (5 copies/μL-100 copies/μL) for each targeted pathogen were tested and 10 replicates were used for each dose. LOD of the PM-TAC was defined as the lowest concentration of the control plasmid that could be detected in all of the 10 replicated samples for each targeted pathogen. The sensitivity and specificity of the PM-TAC were estimated using ACSF spiked with a pure culture of *Streptococcus pneumoniae*, *Streptococcus agalactiae, Staphylococcus aureus*, *Listeria monocytogenes, Staphylococcus epidermidis*, *Pseudomonas aeruginosa*, *Acinetobacter baumannii, Escherichia coli*, *Klebsiella pneumoniae*, or *Cryptococcus neoformans*. Twenty replicates were used for each spiked ACSF. The volume of each spiked ACSF was 500 μL. Total nucleic acid extraction method for clinical CSF samples was used for the spiked ACSF samples. A real-time PCR was used as a gold standard to estimate the sensitivity and specificity.

### Cerebrospinal fluid samples

CSF samples were obtained from the Department of Infectious Disease of Beijing Children’s Hospital (Additional file 1: Table S2). The diagnosis of PM followed the WHO diagnostic guidelines. WHO writes that a suspected PM case with CSF examination showing at least one of the following: turbid appearance; leukocytosis (> 100 cells/mm^3^); leukocytosis (10–100 cells/mm^3^) AND either elevated protein (> 100 mg/dL) or decreased glucose (< 40 mg/dL). A total of 32 children with PM were selected with a representative cross-section of the patient population in the Department of Infectious Disease of Beijing Children’s Hospital. Of the 32 patients, 13 showing positive CSF culture before receiving antibiotics but negative after receiving antibiotics, 2 showing positive CSF culture before antibiotic therapies and remaining positive after the antibiotic therapies, 17 showing negative CSF culture before and after antibiotic therapies. All of the 32 children presented at least one typical clinical symptoms of PM, such as intermittent fever, headache, anterior fontanelle full, vomiting, convulsions, mental changes, altered consciousness, and meningeal irritation. Ten children without PM were also selected to evaluate the specificity of the PM-TAC. The ten children presented fever and similar neurologic symptoms such as meningeal irritation sign. In order to rule out PM, CSF examination was done. And CSF examination of this ten children was normal according to the WHO guidelines.

### Total nucleic acid extraction

ACSF spiked with different concentrations of *Streptococcus pneumoniae* or *Cryptococcus neoformans* culture was used to optimize the nucleic acid extraction method*.* The modified protocol of QIAamp cador pathogen Mini Kit was used. Briefly, 250 μL of each sample was added to the pathogen lysis tube, which contained 0.2 g glass beads with a particle size of 450 μm to 600 μm and 0.2 g glass beads with a particle size of 100 μm (Sigma, St. Louis, MO). The lysis tube was centrifuged for 5 min at 14,000×g, and then 200 μL of the supernatant was transferred to a 1.5 mL microcentrifuge tube containing 20 μL QIAGEN Protease K. A total of 400 μL lysis buffer was added into the pathogen lysis tube to re-suspend the pellet. The pathogen lysis was then extracted as the manual. Total nucleic acid was eluted in 100 μL AVE buffer. A blank control (H_2_O) was included for each batch of extraction to monitor laboratory contaminations. If the blank control showed positive on the PM-TAC assay, the entire batch of total nucleic acid was discarded.

### PM-TAC assay

A total of 50 μL total nucleic acid was used to prepare a reaction mixture (a total volume of 100 μL) for the PM-TAC assay. The ABI master mix (Art.No.4440038) was used. The PM-TAC has 8 channels, 7 for samples and one for a blank control. The reaction mixture was loaded into the inlet port of each channel. The PM-TAC was then centrifuged twice at 1200 rpm for 1 min and sealed. After the centrifugation, the inlet ports were removed according to the manufacturer’s instruction. The PM-TAC was analyzed on the Vii A 7 real-time PCR system (Life Technologies) using the following PCR cycling conditions: 50 °C for 2 min and 95 °C for 10 min, 40 cycles of 95 °C for 15 s and 60 °C for 45 s. A sample was considered positive for the targeted pathogen when the cycle threshold (CT) value was < 38.0 for the PM-TAC assay and the extraction blank control was negative.

### Ethics statement

The study protocols were approved by the Institutional Review Boards of the Institute of Microbiology and Epidemiology and Capital Medical University of China. Written informed consent was obtained from a parent or guardian prior to collect CSF from the children.

## Results

### Performance of each assay on 96-well plate using individual real-time PCR

**Fig. 3:**
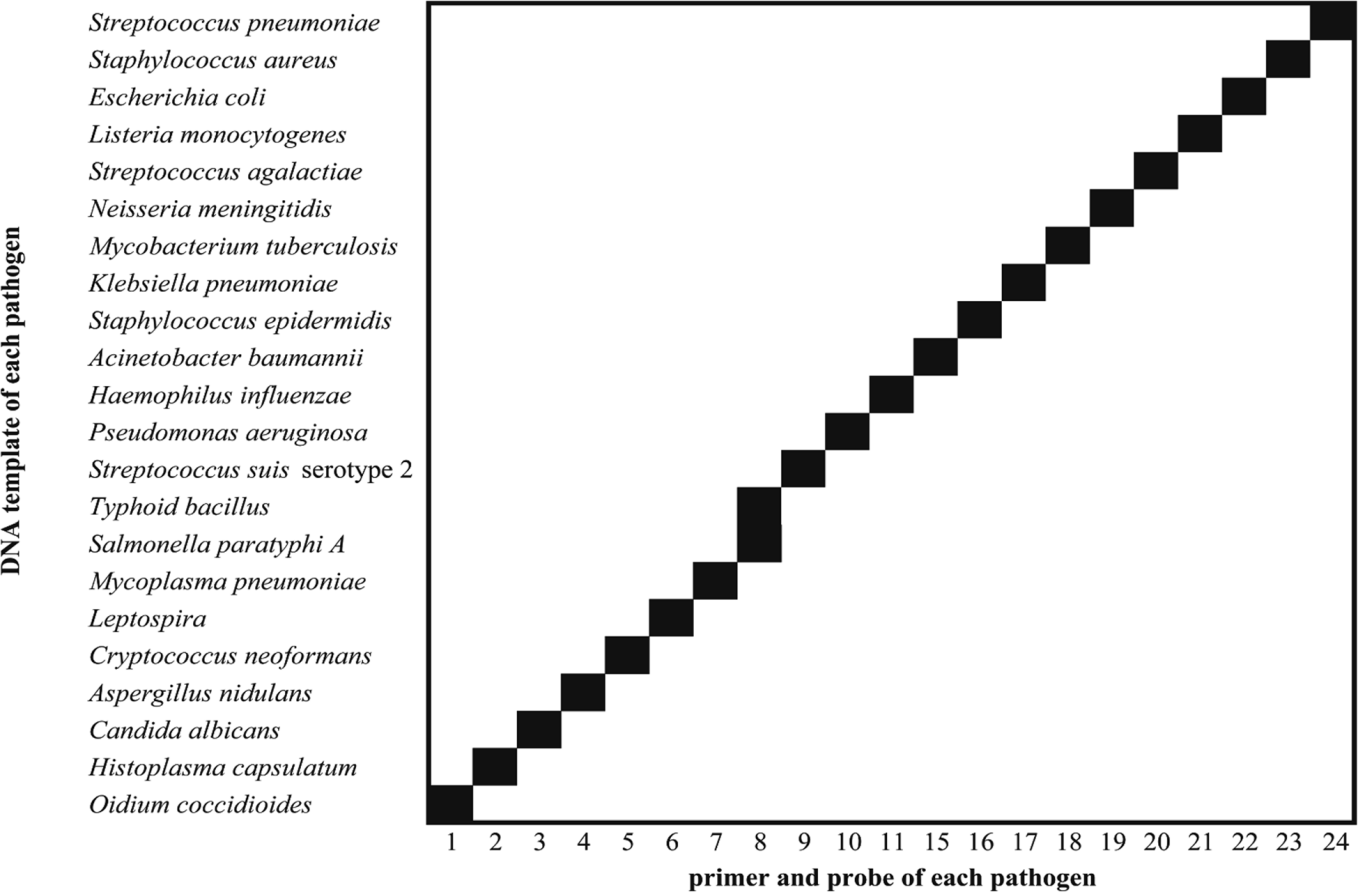
The cross-reactivity of the primers and probes on Purulent Meningitis-TaqMan Array Card. The cross-reactivity of the primers and probes was tested on 96-well plates. Off-target DNA templates, which do not contain the DNA temple sequence for a specific primer/probe pair, were added into the reaction mix to analyze the cross-reactivity of each primer and probe pair. The amplification shows a very high specificity. Only on-target DNA template, which only contains the DNA template sequence for a specific primer/probe pair, was amplified by each specific primer and probe pair

**Fig. 4:**
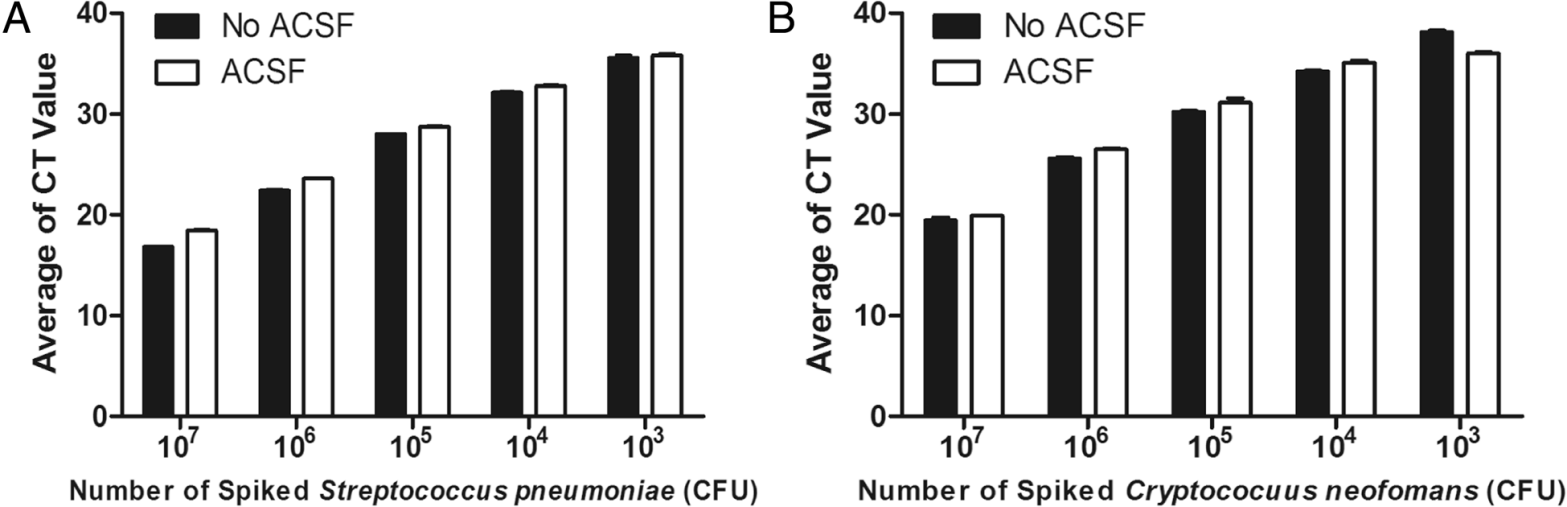
Analysis of matrix inhibition of the extraction of artificial cerebrospinal fluid. **a.** ACSF spiked with *Streptococcus pneumoniae*. **b.** ACSF spiked with *Cryptococcus neoformans.* Different concentrations of the bacteria were added in the ACSF. ACSF had no significant effects on the cycle threshold values

Real-time PCR was first performed to validate the primers and probes using a 96-well plate. The control plasmid was used as the template in the real-time PCR for the validation. For each primer and probe pair, the CT value and the template concentration showed a significant linear correlation. For the 21 targeted pathogens, linear correlation coefficient (R^2^) ranged from 0.995 to 1.0; PCR efficiency ranged from 82.8 to 96.7%; LOD ranged from 3.125copies/μL to 100 copies/μL (Table 1). The cross-reactivity of the primers and probes was also tested on 96-well plates. Off-target DNA templates, which do not contain the DNA temple sequence for a specific primer/probe pair, were added into reaction mix to analyze the cross-reactivity of each primer and probe pair. The amplification showed a very high specificity (Fig. 3). Only the on-target DNA template, which only contains the DNA template sequence for a specific primer/probe pair, was amplified by each specific primer and probe pair (Fig. 3). ACSF spiked with different concentrations of *Streptococcus pneumoniae* or *Cryptococcus neoformans* culture was used to evaluate the nucleic acid extraction method*.* ACSF had no effects on CT values, indicating that the matrix inhibitors in ACSF, such as divalent salts and urea, appeared to be removed effectively during total nucleic acid extraction (Fig. 4).

**Table 1:** Evaluation of primers and probes using real-time PCR

	R^2^	Efficiency (%)	LOD (copies/μL)
*Streptococcus pneumoniae*	1.0	90.23	100
*Staphylococcus aureus*	0.998	87.12	25
*Escherichia coli*	0.999	90.12	100
*Listeria monocytogenes*	1.0	94.42	25
*Streptococcus agalactiae*	0.997	85.42	25
*Neisseria meningitidis*	1.0	96.46	25
*Mycobacterium tuberculosis* complex	0.999	97.59	25
*Leptospira*	0.999	84.55	6.25
*Mycoplasma pneumoniae*	0.999	89.6	10
*Oidium coccidioides*	0.999	89.78	3.125
*Histoplasma capsulatum*	1.0	92.76	6.25
*Cryptococcus neoformans*	0.999	92.75	6.25
*Staphylococcus epidermidis*	0.997	92.75	100
*Acinetobacter baumannii*	0.995	85.81	100
*Streptococcus suis* serotype 2	0.999	85.0	12.5
*Pseudomonas aeruginosa*	1.0	85.36	50
*Candida albicans*	0.997	82.8	5
*Haemophilus influenzae*	0.998	94.1	5
*Klebsiella pneumoniae*	0.999	96.7	3.125
*Aspergillus nidulans*	0.997	95.0	3.125
*Salmonella spp.*	0.998^a^/0.995^b^	90.0^a^/92.1^b^	10^a^/25^b^

*Salmonella spp.*in this study included *Typhoid bacillus* (a) and *Salmonella paratyphi A* (b)

### Performance for evaluation of PM-TAC

We also evaluated the primers and probes in the PM-TAC assay. ACSF was spiked with the control plasmid first, and then the ACSF containing the control plasmid was used for the validation of the primers and probes in the PM-TAC assay. In the PM-TAC assay, for the 21 targeted pathogens, the linear correlation coefficient (R^2^) ranged from 0.99 to 0.999; PCR efficiency was greater than 88.469%; LOD ranged from 5 copies/reaction to 50copies/reaction except *Streptococcus pneumoniae* with a LOD of 100 copies/reaction. The intra-assay variation of the PM-TAC assay at high and low concentration of the template was ≤1.84% and ≤ 4.45%, respectively, and the inter-assay variation at the high and low concentration of the template was ≤5.21% and ≤ 6.81%, respectively, for all of the target pathogens (Additional file 1: Table S3). We evaluated the sensitivity and specificity of the PM-TAC using ACSF spiked with the targeted pathogens. The overall sensitivity and specificity were 95 and 96%, respectively (Table 2).

**Table 2:** Sensitivity and specificity of the PM-TAC assay using ACSF spiked with targeted pathogens

	Numbers of samples			
Targeted Pathogen	IRTP + and PM-TAC +	IRTP + and PM-TAC -	IRTP - and PM-TAC +	IRTP - and PM-TAC -
*Streptococcus pneumoniae*	18	2	0	10
*Streptococcus agalactiae*	19	1	0	10
*Staphylococcus aureus*	20	0	1	9
*Listeria monocytogenes*	19	1	1	9
*Staphylococcus epidermidis*	18	2	0	10
*Pseudomonas aeruginosa*	19	1	2	10
*Klebsiella pneumoniae*	20	0	0	9
*Acinetobacter baumannii*	20	0	0	10
*Escherichia coli*	20	0	0	10
*Cryptococcus neoformans*	17	3	0	10
Total	Sensitivity = 95%		Specificity = 96%	

*ACSF* artificial cerebrospinal fluid, *IRTP* Individual Real-time PCR. IRTP was used as the gold standard

### Comparison of PM-TAC versus the CSF culture method

Only two of the 15 CSF samples showing positive CSF culture before antibiotic therapies remained positive CSF culture after the antibiotic therapies. In contrast to the CSF culture results, 8 of the 15 samples showed positive from the PM-TAC assay after antibiotic therapies (Table 3). We conducted 16SrDNA PCR sequencing to confirm the PM-TAC results. The results of PCR/sequencing and PM-TAC were consistent (Table 3). We used 17 CSF samples of PM showing negative CSF culture before and after antibiotic therapies to further evaluate the sensitivity of the PM-TAC (Additional file 1: Table S4). Of the 17 samples, one was positive for *Neisseria meningitidis* from the PM-TAC assay (confirmed by PCR sequencing) (Additional file 1: Table S4). Notably, one sample was negative from both CSF culture and PM-TAC assay but positive for *Enterococcus faecium* from the PCR/sequencing analysis (Additional file 1: Table S4). The reason for this inconsistency was that the pathogen *Enterococcus faecium* was not included on the PM-TAC in this study. To further evaluate the specificity of the PM-TAC, we used 10 CSF samples from patients without central nervous system infection. The 10 CSF samples showed negative from both the PM-TAC assay and the CSF culture method (Additional file 1: Table S5). These data demonstrated that the specificity of the PM-TAC was 100%.

**Table 3:** Comparison of the PM-TAC assay versus the CSF culture method

Patients No.	CSF culture result		PM-TAC assay result	Concordance	PCR/sequencing result
	Before antibiotic therapies	After antibiotic therapies	After antibiotic therapies		
001	*Streptococcus pneumoniae*	Negative	Negative	Yes	Negative
002	*Streptococcus pneumoniae*	Negative	*Streptococcus pneumoniae*	No	*Streptococcus* pneumoniae
003	*Klebsiella pneumoniae*	*Klebsiella pneumoniae*	*Klebsiella pneumoniae*	Yes	–
004	*Haemophilus influenzae*	Negative	Negative	Yes	Negative
005	*Klebsiella pneumoniae*	Negative	*Klebsiella pneumoniae*	No	*Klebsiella pneumoniae*
006	*Escherichia coli*	Negative	*Escherichia coli*	No	*Escherichia coli*
007	*Salmonella spp*	Negative	Negative	Yes	Negative
008	*Baumanii*	*Baumanii*	*Baumanii*	Yes	–
009	*Streptococcus pneumoniae*	Negative	Negative	Yes	Negative
010	*Streptococcus pneumoniae*	Negative	Negative	Yes	Negative
011	*Streptococcus pneumoniae*	Negative	*Streptococcus pneumoniae*	No	*Streptococcus pneumoniae*
012	*Escherichia coli*	Negative	*Escherichia coli*	No	*Escherichia coli*
013	*Escherichia coli*	Negative	*Escherichia coli*	No	*Escherichia coli*
014	*Streptococcus pneumoniae*	Negative	Negative	Yes	Negative
015	*Listeria monocytogenes*	Negative	Negative	Yes	Negative

### The association of positive PM-TAC results and PM severity

To evaluate whether PM-TAC results could indicate PM severity, we analyzed the cycle threshold (CT) of PM-TAC assay and patient clinical presentations. A lower CT of PM-TAC assay represents higher pathogen loads in samples. We compared CT and clinical presentation in patients showing positive for the same pathogen (Table 4). For the two patients showing positive *Streptococcus pneumoniae* in CSF, the patient with a lower CT (34.0) presented intermittent fever for 4 months, higher CSF cell count, and bilateral subdural effusion and left subdural empyema, whereas the patient with a high CT (37.72) did not present those complications. Similarly, the patient with a low CT (30.72) of *Klebsiella pneumoniae* presented more severe symptoms and complications, such as cerebral ventricle inflammation, subdural effusion and empyema, and hearing loss, compared with the patient with a higher CT (34.49), who only presented subdural effusion. Consistently, in the three patients showing positive *Escherichia coli* from the PM-TAC assay, the one with the lowest CT (19.71) presented recurrent seizures and subdural empyema and with mass effect and developed hemiplegic paralysis, whereas the other two patients showed high CTs (33.34 and 33.59) presented milder complications and no post-PM sequelae (Table 4).

**Table 4:** The pathogen load in CSF samples and the severity of PM

Patients No.	Pathogen	CT value	Complications	Sequelae
002	*Streptococcus pneumoniae*	34.0	Bilateral subdural effusion and left subdural empyema	None
011	*Streptococcus pneumoniae*	37.72	None	None
003	*Klebsiella pneumoniae*	30.72	Subdural effusion and empyema, cerebral ventricle inflammation, hearing loss	Hearing loss
005	*Klebsiella pneumoniae*	34.49	Subdural effusion	None
006	*Escherichia coli*	19.71	Subdural empyema with mass effect	Hemiplegic paralysis recurrent seizures
012	*Escherichia coli*	33.34	Subdural effusion	None
013	*Escherichia coli*	33.59	Hydrocephalus	None

*CT* Cycle threshold

## Discussion

In the current study, we developed a PM-TAC and optimized the PM-TAC assay. Our PM-TAC showed a LOD of 5 to 50 copies/reaction, except *Streptococcus pneumoniae* with a LOD of 100 copies/reaction, a sensitivity of 95%, and a specificity of 96%. These performance parameters are similar to the findings from a previous study [11]. Compared with the CSF culture method, which usually has a sensitivity of 81.3%, our PM-TAC was more sensitive [7, 10]. In addition to the superior sensitivity, our PM-TAC assay produced results fairly faster (in 3 h) at a lower cost ($126/sample) than the next generation sequencing (NGS) method (72 h of the assay time and $500/sample) [14]. Although the NGS technology has been improved continuously, it remains to be tedious and requires comprehensive bioinformatic analyses. All of those disadvantages of the NGS method limit its application in clinical practice. In contrast to the NGS method, it only took 3 h to collect results from our PM-TAC assay. Liu and colleagues have shown that the cost of TAC assay is much lower than that of ELISA and other methods [28] The estimated average cost of our PM-TAC assay for each targeted pathogen was approximately $6 and $144 for a whole panel ($6 x [21 PM-associated pathogens+ 3 controls] = $144), which appears affordable for economically underdeveloped regions and countries.

Compared with the CSF culture method, our PM-TAC assay was particularly more sensitive to detect pathogens after empiric antibiotic therapies. The positive rate of our PM-TAC was 53.3% (8/15), whereas it was only 13.3% (2/15) for the CSF culture method for CSF samples collected after empiric antibiotic therapies. These results indicate substantial clinical significance. The PM-TAC assay allows for detection of these pathogens which is independent of the use of antibiotics. The assay can then be used to assess the efficacy of antibiotic therapy in clearing the pathogen and the infection. Previous studies have demonstrated that the rate of inappropriate antibiotic use in the hospital setting is 30 to 50% because of the lacking an accurate identification of the causative bacteria [31, 32]. Every pathogen is sensitive to the specific antibiotics. The inappropriate antibiotic use before the pathogen was identified could induce drug resistant in bacteria. To be clinically effective, antibiotics that are specifically targeting the causative bacteria should be prescribed at the proper dosage.

In addition, we found that the CT of a pathogen from the PM-TAC assay appeared to indicate PM severity. Primary infections are usually pathogenic specific, and thus a pathogen load is associated with the infection severity. Our findings suggest that the PM-TAC assay could provide an effective reference to guide the therapeutic strategies for PM. For example, clinicians could adjust the dosage of antibiotic treatments bases on the PM-TAC results. Our PM-TAC system also allows multiplex detection. According to the clinical experiences of pediatricians, most cases of PM are caused by a single pathogen. In our current study, the 15 clinical CSF samples also showed a single-pathogen infection, which confirmed the clinical experiences.

There are limitations in our current study. There was a small sample size indeed in this study due to the difficulty to collect enough CSF in children clinically. Because many clinical examinations were performed so almost all cerebrospinal fluid were used up and this reduced greatly the sample size. Although the sample size was small, it could reflect a very meaningful phenomenon. Fortunately, we constructed simulated CSF samples being close to the clinical CSF sample based on previously reported studies [33], which made up for the small sample size. Moreover, we are also making efforts to collect new CSF samples from hospitals, but it will take a long time, and with the accumulation of sample size of CSF, we plan to do more in-depth related research in the future. The cost of PM-TAC is still prohibitively expensive for resource poor settings although it is cheaper compared with other molecular diagnostic techniques. Actually, the main cost is the microfluidic chip and at present, microfluidic versions have great potential applications in all aspects and have attracted much attention. At the same time, the production technology of microfluidic plate is developing rapidly. When the material and technology of TAC break through, the cost of this method will be further reduced. In addition, we are also increasing the number of pathogen detected, so that the detection cost of each pathogen will be lower. All of the targeted pathogens on the PM-TAC were selected mainly based on common clinical experiences. Therefore, the PM-TAC is unable to identify previously unidentified pathogens whereas the NGS method could. We will continuously update and expand the targeted pathogen panel on the PM-TAC following the most updated literature. Although the evolutionarily conserved domains were selected to target the pathogens, mutations in the targeted gene may cause false negative results because of the high specificity of the minor groove binder probes. In general, pathogenic bacteria and fungi have a low mutation rate, whereas virus often mutate quickly. Our future study will take gene mutations into consideration when we design a PM-TAC.

## Conclusions

The PM-TAC in this study can detect 21 PM-associated pathogens in 3 h and showed a higher sensitivity than the CSF culture method particularly for CSF samples collected after empiric antibiotic therapies. The CT of a pathogen from the PM-TAC assay, which represents a pathogen concentration or load in CSF samples, indicated PM severity. Thus, the PM-TAC may be a promising tool for an accurate and rapid diagnosis of PM and thus facilitate a timely antibiotic treatment to improve PM prognosis.

## Additional file

Additional file 1: **Table S1.** Primer and probe sequences of the 21 targeted pathogens. **Table S2.** The information about the 32 patients. **Table S3.** Validation of the primers and probes in the PM-TAC assay using ACSF spiked with the control plasmid. **Table S4.** Comparison of the PM-TAC assay versus the CSF culture method on samples showing negative CSF culture from PM patients. **Table S5.** Comparison of the PM-TAC assay versus the CSF culture method on CSF samples from patients who are not PM patients [34–45]. (DOCX 39 kb)

## Abbreviations

ACSF: artificial cerebrospinal fluid
CNS: central nervous system
CSF: conventional cerebrospinal fluid
CT: cycle threshold
NGS: the next generation sequencing
PM: Purulent meningitis
PM-TAC: purulent meningitis-TaqMan array card
TAC: TaqMan array card
