# Supplementary material for: Development of a TaqMan Array card to target 21 purulent meningitis-related pathogens
Source: BMC Infect Dis. 2019 Mar 28;19:289. doi: 10.1186/s12879-019-3856-z (PMC6438039; doi:10.1186/s12879-019-3856-z)
Supplement: Supplementary file 1 — Table S1. Primer and probe sequences of the 21 targeted pathogens. Table S2. The information about the 32 patients. Table S3. Validation of the primers and probes in the PM-TAC assay using ACSF spiked with the control plasmid. Table S4. Comparison of the PM-TAC assay versus the CSF culture method on samples showing negative CSF culture from PM patients. Table S5. Comparison of the PM-TAC assay versus the CSF culture method on CSF samples from patients who are not PM patients [34–45]. (DOCX 39 kb) [file 12879_2019_3856_MOESM1_ESM.docx]

**Table S1 Primer and probe sequences of the 21 targeted pathogens**

| Pathogen | Target gene | Sequence | Product length | Reference |
| --- | --- | --- | --- | --- |
| *Klebsiella pneumoniae* | *Diguanylate cyclase* | F:TGCAGATAATTCACGCCCAG | 133bp | Modified[26] |
|  |  | R:ACCCGCTGGACGCCAT |  |  |
|  |  | P:CGCTCATCTGTTTCGC |  |  |
| *Neisseria meningitidis* | *ctrA* | F:GCTGCGGTAGGTGGTTCAA | 111bp | Modified[34] |
|  |  | R:TTGTCGCGGATTTGCAACTA |  |  |
|  |  | P:ACGTGTCAGCTGCACAT |  |  |
| *Streptococcus pneumoniae* | *ply* | F:TGCAGAGCGTCCTTTGGTCTAT | 81bp | Modified[34] |
|  |  | R:CTCTTACTCGTGGTTTCCAACTTGA |  |  |
|  |  | P:CCCATAAGCAACACTCGAA |  |  |
| *Mycobacterium tuberculosis* complex | *IS6110* | F:GGCTGTGGGTAGCAGACC | 163bp | Modified[35] |
|  |  | R:CGGGTCCAGATGGCTTGC |  |  |
|  |  | P:ACCTGGGCAGGGTTCG |  |  |
| *Staphylococcus aureus* | *Glutamate synthase* | F:CGGGTTAGGTGAATTGATTGTTTTAT | 130bp | Modified[26] |
|  |  | R:CGCATTTGAGCTGAAGTTG |  |  |
|  |  | P:ACCACGAGTCTTAGCACC |  |  |
| *Escherichia coli* | *uidA* | F:GAGCATCAGGGTGGCTATACG | 113bp | Modified[26] |
|  |  | R:ATAGTCTGCCAGTTCAGTTC |  |  |
|  |  | P:CGTGACATCGGCTTCAAAT |  |  |
| *Listeria monocytogenes* | *hly* | F:TTTCATCCATGGCACCACC | 71bp | Modified[36] |
|  |  | R:ATCCGCGTGTTTCTTTTCGA |  |  |
|  |  | P:TGCAAGTCCTAAGACGCCA |  |  |
| *Streptococcus agalactiae* | *cfb* | F:GGGAACAGATTATGAAAAACCG | 126bp | Modified[26] |
|  |  | R:AAGGCTTCTACACGACTACCAA |  |  |
|  |  | P:CGTGCCAACCCTGAGAC |  |  |
| *Haemophilus influenzae* | *bexA* | F:GGACAAACATCACAAGCGGTTA | 116bp | Modified[22] |
|  |  | R:TGCGGTAGTGTTAGAAAATGGTATTATG |  |  |
|  |  | P:TTGTAGTATTGATACGCTTTGT |  |  |
| *Streptococcus suis* serotype 2 | *cps2J* | F:GAGGGTTACTTGCTACTTTTGATG | 107 | [37] |
|  |  | R:ATTTTCATTTCCTAAGTCTCGCACC |  |  |
|  |  | P:TTGGAAGAGATAAAAGAGG |  |  |
| *Staphylococcus epidermidis* | *tpi* | F:CATCTGATAAACCTTCGACAGCTTT | 128bp | Modified[38] |
|  |  | R:TGCTATCTTCAATCACGGTATGAC |  |  |
|  |  | P:TTCACGTTCTTCATCAGATT |  |  |
| *Acinetobacter baumannii* | *ompA* | F:AGTTCTTGGTGGTCACTTGAAGC | 92bp | Modified[39] |
|  |  | R:TTAACTCTTGTGGTTGTGGAGCA |  |  |
|  |  | P:AGTTGAACCAACTCCA |  |  |
| *Pseudomonas aeruginosa* | *23srRNA* | F:TCCAAGTTTAAGGTGGTAGGCTG | 94bp | Modified[43] |
|  |  | R:ACCACTTCGTCATCTAAAAGACGAC |  |  |
|  |  | P:TAAATCCGGGGTTTCAAGGCC |  |  |
| *Mycoplasma pneumoniae* | *P1* | F:GCAGTTGCTGGCGCTAAGTT | 86bp | Beijing genomics constitution |
|  |  | R:AAGCGAGGTACGGTAGCGGTAT |  |  |
|  |  | P:TGGTAGGGAACTCGTTTTA |  |  |
| *Leptospira* | *lipL32* | F:AAGCATTACCGCTTGTGGTG | 242bp | Modified[41] |
|  |  | R:GAACTCCCATTTCAGCGATT |  |  |
|  |  | P:AGCCAGGACAAGCGCCG |  |  |
| *Candida albicans* | *26s* | F:CTTGGTATTTTGCATGYTGCTCTC | 128bp | Modified[42] |
|  |  | R:GTCAGAGGCTATAACACACAGCAG |  |  |
|  |  | P:TGCGTTTACCGGGCCA |  |  |
| *Cryptococcus neoformans* | *5.8S and ITS* | F:CCTGTTGGACTTGGATTTGG | 140bp | Modified[43] |
|  |  | R:AGCAAGCCGAAGACTACC |  |  |
|  |  | P:CAGGTAATCAGATCGCG |  |  |
| *Salmonella spp* | *invA* | F:TCGGGCAATTCGTTATTGG | 77bp | Modified[44] |
|  |  | R:GATAAACTGGACCACGGTGACA |  |  |
|  |  | P:AGACAACAAAACCCACCGC |  |  |
| *Histoplasma capsulatum* | *5.8S and ITS* | F:GTCTGAGCATGAGAGCGATAATAATC | 83bp | This study |
|  |  | R:CGCTGCGTTCTTCATCGA |  |  |
|  |  | P:CTTTCAACAACGGATCTC |  |  |
| *Oidium coccidioides* | *5.8S and ITS* | F:CGTACCTCCCACCCGTGTT | 195bp | This study |
|  |  | R:AAGAGATCCGTTGTTGAAAGTTTTG |  |  |
|  |  | P:TATGTGAAGATTGTCAGTCTG |  |  |
| *Aspergillus nidulans* | *5.8s and ITS* | F:CTGTCCGAGCGTCATTG | 240bp | Modified[45] |
|  |  | R:TCCTCCGCTTATTGATAT |  |  |
|  |  | P:CACCCGCTCGATTAGG |  |  |

F: forward primer; R: reverse primer; P: probe, labeled with FAM (6-carboxyfluorescein) at 5’ and MGB at 3’.

**Table S2** **The information about the 32 patients**

| Patients No. | Symptoms and signs | | | Biochemical, and cytological data | | |
| --- | --- | --- | --- | --- | --- | --- |
|  | Fever(℃) | Consciousness | Meningeal irritation sign | Protein(mg/dL) | Glucose(mg/dL) | WBCs(total/mm^3^) |
| 001 | Yes (39) | coma | Nuchal rigidity and Brudzinski | 702 | 1.44 | 3210 |
| 002 | Yes (39.7) | drowsiness | Nuchal rigidity | 0.1 | 37.98 | 9999 |
| 003 | Yes (39) | drowsiness | Nuchal rigidity | 313.8 | 4.32 | 17810 |
| 004 | Yes (39) | drowsiness | - | 243 | 19.8 | 6000 |
| 005 | Yes (39.2) | awake | - | 400 | 11.52 | 13 |
| 006 | Yes (39.1) | drowsiness | Nuchal rigidity | 300 | 9.36 | 5480 |
| 007 | Yes (38.6) | drowsiness | - | 284.2 | 3.6 | 750 |
| 008 | Yes (41) | coma | Nuchal rigidity | 200 | 54 | 46390 |
| 009 | Yes (39.8) | awake | - | 212.1 | 3.6 | 1990 |
| 010 | Yes (42) | drowsiness | Nuchal rigidity, Kernig and Brudzinski | 196 | 36 | 450 |
| 011 | Yes (40) | awake | - | 227 | 0.36 | 46 |
| 012 | Yes (39) | awake | Nuchal rigidity | 134 | 36 | 760 |
| 013 | Yes (38.3) | drowsiness | - | 775 | 40.14 | 3100 |
| 014 | Yes (38.8) | awake | - | 600 | 9 | 1840 |
| 015 | Yes (40) | awake | Nuchal rigidity | 111.6 | 74.34 | 2310 |
| 016 | Yes (40) | drowsiness | Nuchal rigidity | 100.9 | 54.9 | 135 |
| 017 | Yes (38.8) | drowsiness | - | 155.3 | 46.08 | 186 |
| 018 | Yes (39.5) | awake | Nuchal rigidity, Kernig and Brudzinski | 124.9 | 17.82 | 1080 |
| 019 | Yes (39) | drowsiness | Nuchal rigidity | 166 | 10.08 | 260 |
| 020 | Yes (38.5) | awake | Nuchal rigidity | 200 | 18 | 210 |
| 021 | Yes (40) | coma | Nuchal rigidity | 274 | 7.2 | 1539 |
| 022 | Yes (39) | awake | - | 51 | 75.42 | 260 |
| 023 | Yes (39.1) | coma | Nuchal rigidity | 471.5 | 8.46 | 330 |
| 024 | Yes (38) | awake | Nuchal rigidity | 270 | 49.86 | 1600 |
| 025 | Yes (39.7) | awake | - | 12 | 63 | 200 |
| 026 | Yes (40.2) | awake | - | 210 | 19.08 | 172 |
| 027 | Yes (39.4) | drowsiness | Nuchal rigidity | 999.5 | 86.76 | 1460 |
| 028 | Yes (38.9) | awake | - | 73.9 | 45 | 180 |
| 029 | Yes (41) | coma | Nuchal rigidity, Kernig and Brudzinski | 328 | 23.76 | 1850 |
| 030 | Yes (38.9) | awake | - | 385 | 21.78 | 27200 |
| 031 | Yes (38.5) | drowsiness | Nuchal rigidity | 265.3 | 108 | 760 |
| 032 | Yes (38.6) | awake | - | 192.5 | 2.7 | 960 |

**Table S3 Validation of the primers and probes in the PM-TAC assay using ACSF spiked with the control plasmid**

|  | Linearity | |  | Intra-assay variation (%) | | Inter-assay variation (%) | |
| --- | --- | --- | --- | --- | --- | --- | --- |
| Targeted Pathogen | R^2^ | Efficiency % | LOD (copies/reaction) | High concentration | Low concentration | High concentration | Low concentration |
| *Streptococcus pneumoniae* | 0.991 | 98.04 | 100 | 1.50 | 0.71 | 0.57 | 6.09 |
| *Staphylococcus aureus* | 0.996 | 100.64 | 50 | 0.57 | 0.78 | 0.54 | 5.54 |
| *Escherichia coli* | 0.996 | 102.63 | 5 | 0.07 | 1.33 | 0.73 | 4.95 |
| *Listeria monocytogenes* | 0.99 | 104.31 | 5 | 0.47 | 1.11 | 1.20 | 2.75 |
| *Streptococcus agalactiae* | 0.997 | 105.21 | 50 | 0.26 | 0.68 | 2.46 | 5.83 |
| *Neisseria meningitidis* | 0.997 | 102.72 | 50 | 0.28 | 1.80 | 1.32 | 6.06 |
| *Mycobacterium tuberculosis* complex | 0.996 | 99.32 | 50 | 0.61 | 2.53 | 1.95 | 2.54 |
| *Leptospira* | 0.998 | 88.469 | 50 | 0.19 | 2.82 | 2.29 | 1.63 |
| *Mycoplasma pneumoniae* | 0.999 | 91.258 | 25 | 0.41 | 3.54 | 0.87 | 4.08 |
| *Oidium coccidioides* | 0.998 | 95.014 | 10 | 1.34 | 1.20 | 1.29 | 0.13 |
| *Histoplasma capsulatum* | 0.996 | 97.513 | 10 | 0.89 | 0.58 | 1.60 | 3.13 |
| *Cryptococcus neoformans* | 0.998 | 103.56 | 10 | 0.30 | 2.24 | 5.17 | 0.00 |
| *Staphylococcus epidermidis* | 0.997 | 100.01 | 25 | 1.84 | 1.26 | 0.77 | 6.81 |
| *Acinetobacter baumannii* | 0.998 | 96.71 | 25 | 0.21 | 1.22 | 1.59 | 3.88 |
| *Streptococcus suis* serotype 2 | 0.999 | 95.43 | 50 | 0.27 | 0.55 | 1.27 | 2.29 |
| *Pseudomonas aeruginosa* | 0.995 | 101.98 | 10 | 0.59 | 1.34 | 5.21 | 1.41 |
| *Candida albicans* | 0.996 | 100.34 | 10 | 0.12 | 0.52 | 0.53 | 2.38 |
| *Haemophilus influenzae* | 0.994 | 90.976 | 50 | 0.96 | 0.53 | 0.36 | 1.81 |
| *Klebsiella pneumoniae* | 0.999 | 91.986 | 50 | 0.96 | 4.45 | 1.09 | 0.11 |
| *Aspergillus nidulans* | 0.998 | 92.98 | 5 | 0.90 | 2.10 | 0.88 | 0.97 |
| *Salmonella spp.* | 0.999^a^/0.998^b^ | 93.0^a^/90.3^b^ | 10^a^/10^b^ | 1.01^a^/1.30^b^ | 3.34^a^/3.0^b^ | 2.89^a^/2.50^b^ | 5.79^a^/3.21^b^ |

ACSF: artificial cerebrospinal fluid. Salmonella spp.in this study included *Typhoid bacillus* (a) and *Salmonella paratyphi A* (b).

**Table S4 Comparison of the PM-TAC assay versus the CSF culture method on samples showing negative CSF culture from PM patients**

| **Patients No.** | **CSF culture** | **PM-TAC assay result** | **Concordance** | **PCR/sequencing result** |
| --- | --- | --- | --- | --- |
| 016 | Negative | Negative | Yes | Negative |
| 017 | Negative | Negative | Yes | Negative |
| 018 | Negative | Negative | Yes | Negative |
| 019 | Negative | Negative | Yes | Negative |
| 020 | Negative | Negative | Yes | Negative |
| 021 | Negative | Negative | Yes | Negative |
| 022 | Negative | Negative | Yes | Negative |
| 023 | Negative | Negative | Yes | Negative |
| 024 | Negative | Negative | Yes | Negative |
| 025 | Negative | Negative | Yes | Negative |
| 026 | Negative | Negative | Yes | Negative |
| 027 | Negative | Negative | Yes | Negative |
| 028 | Negative | Negative | Yes | Negative |
| 029 | Negative | *Neisseria meningitidis* | No | *Neisseria meningitidis* |
| 030 | Negative | Negative | Yes | Negative |
| 031 | Negative | Negative | Yes | Negative |
| 032 | Negative | Negative | Yes | *Enterococcus faecium* |

**Table S5 Comparison of the PM-TAC assay versus the CSF culture method on CSF samples from patients who are not PM patients**

| **Patients No.** | **CSF culture** | **PM-TAC** | **Concordance** | **PCR/sequencing result** |
| --- | --- | --- | --- | --- |
| 033 | Negative | Negative | Yes | Negative |
| 034 | Negative | Negative | Yes | Negative |
| 035 | Negative | Negative | Yes | Negative |
| 036 | Negative | Negative | Yes | Negative |
| 037 | Negative | Negative | Yes | Negative |
| 038 | Negative | Negative | Yes | Negative |
| 039 | Negative | Negative | Yes | Negative |
| 040 | Negative | Negative | Yes | Negative |
| 041 | Negative | Negative | Yes | Negative |
| 042 | Negative | Negative | Yes | Negative |

CNS: Central nervous system
